# Supplementary material for: Homology in Sex Determination in Two Distant Spiny Frogs, Nanorana quadranus and Quasipaa yei
Source: Animals (Basel). 2024 Jun 21;14(13):1849. doi: 10.3390/ani14131849 (PMC11240834; doi:10.3390/ani14131849)
Supplement: Supplementary file 1 [file animals-14-01849-s001.zip › Figure S1.pdf]

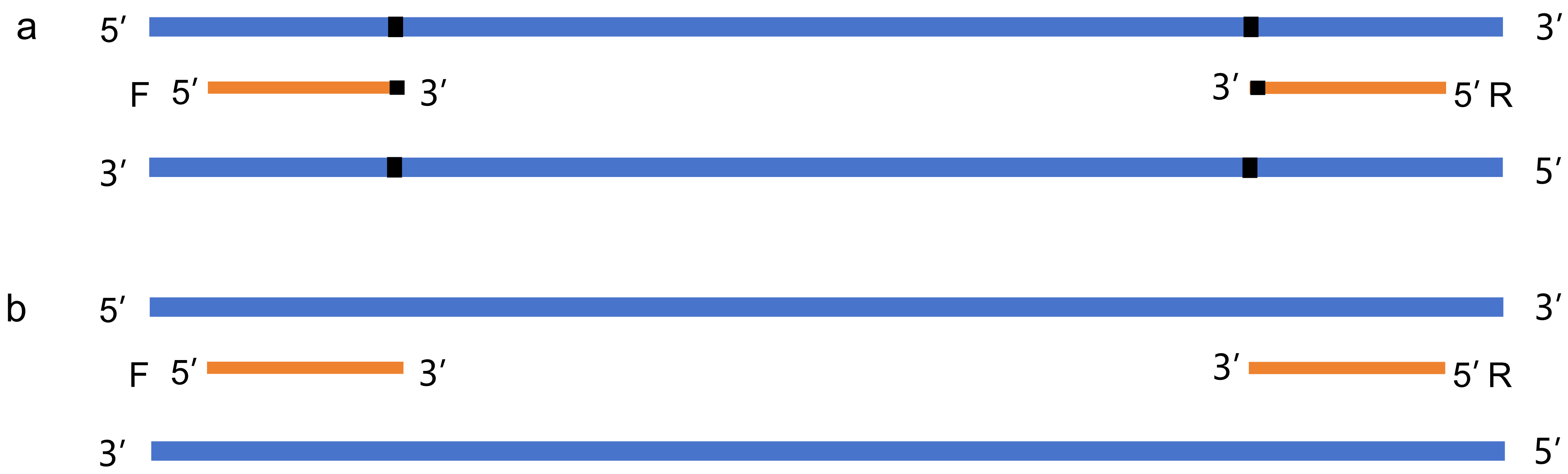

Figure S1. Primer design for sex-linked markers. Examples suggest an XX/XY sex chromosome system, but the results are similar in species with a ZZ/ZW system. Yellow line indicate primers, and 'F' and 'R' indicate forward primer and reverse primer, respectively. Black segments indicate Y-specific SNPs that do not occur on the X. (a) Both the forward and reverse primers were designed at Y-specific SNPs. The Y-specific base was set as the first base of the 3' of the primer sequence. (b) Both of the forward and reverse primers were designed at the male-specific sequence.
