# Supplementary material for: Homology in Sex Determination in Two Distant Spiny Frogs, Nanorana quadranus and Quasipaa yei
Source: Animals (Basel). 2024 Jun 21;14(13):1849. doi: 10.3390/ani14131849 (PMC11240834; doi:10.3390/ani14131849)
Supplement: Supplementary file 1 [file animals-14-01849-s001.zip › Figure S2.pdf]

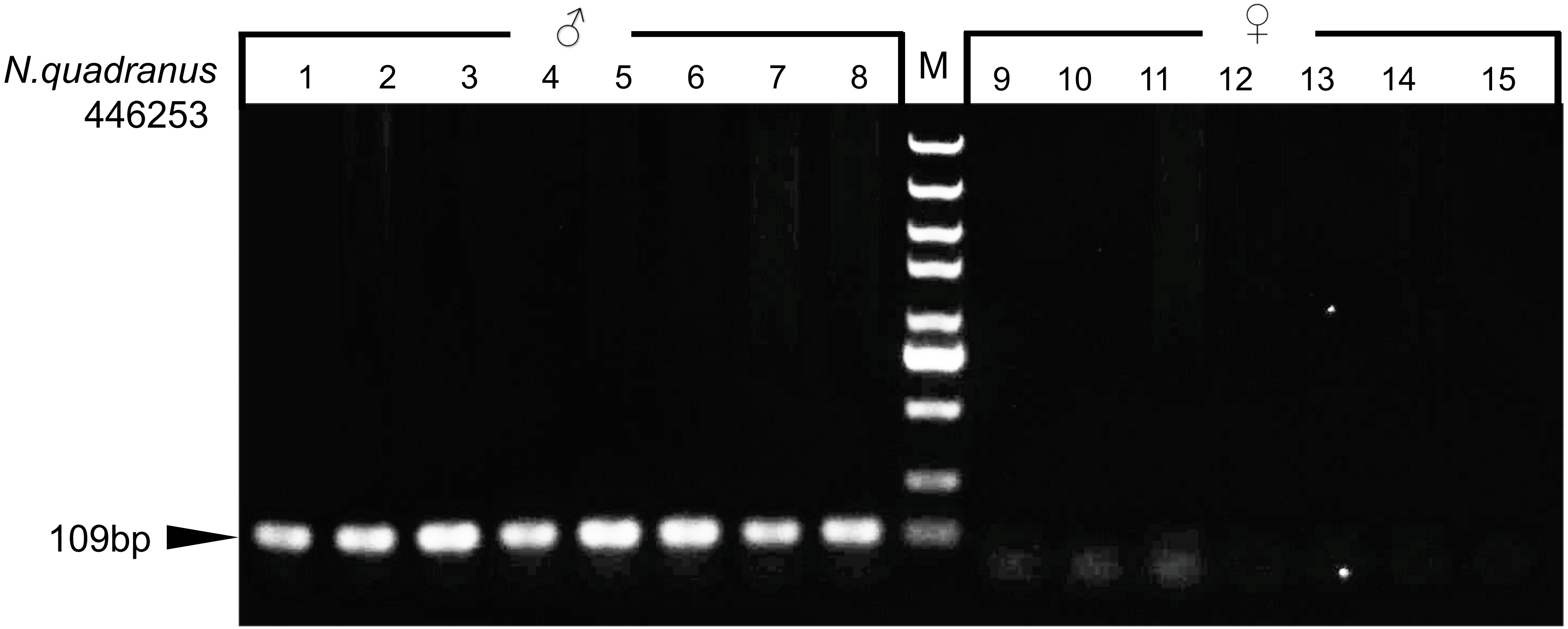


Figure S2. Gel electrophoresis showing the PCR amplification of markers 446253 (*N. quadranus)*. The locus ID is indicated to the left. The symbol ‘ ♂’ representes male individuals and the symbol ‘ ♀’ representes female individuals. ‘ M’ indicates a DNA marker. Black arrows indicate the PCR products size.
