# Supplementary material for: Homology in Sex Determination in Two Distant Spiny Frogs, Nanorana quadranus and Quasipaa yei
Source: Animals (Basel). 2024 Jun 21;14(13):1849. doi: 10.3390/ani14131849 (PMC11240834; doi:10.3390/ani14131849)
Supplement: Supplementary file 1 [file animals-14-01849-s001.zip › File S1.pdf]

Sample size and location information. Male individuals are represented by the symbol “M” and female individuals by the symbol “F”. Specimen number indicated by an underline has been verified by electrophoresis. The voucher specimens were deposited in the Herpetological Museum of Chengdu Institute of Biology, Chinese Academy of Sciences.

1. *Quasipaa Yei* (n=19, 10M, 9F) Gaoniu village, Anhui Province, China (N 31. 418958°，E 115. 403522° )
XM7318M、XM7319M、XM7324M、XM7325M、XM7326M、XM7330M、XM7331M、XM7332M、XM7333M、XM7334M、XM7317F、XM7320F、XM7321F、XM7322F、XM7323F、XM7327F、XM7328F、XM7329F、XYU002F.

2. *Nanorana quadranus* (n=15, 8M, 7F) An village, Sichuan Province, China(N 31. 74315°，E 104. 26106°)
XM3500M、XM3502M、XM3503M、XM3506M、XM3510M、XM3514M、XM3523M、XM3536M、XM3501F、XM3504F、XM3505F、XM3507F、XM3508F、XM3509F、XM3537F.
