# Supplementary material for: Homology in Sex Determination in Two Distant Spiny Frogs, Nanorana quadranus and Quasipaa yei
Source: Animals (Basel). 2024 Jun 21;14(13):1849. doi: 10.3390/ani14131849 (PMC11240834; doi:10.3390/ani14131849)
Supplement: Supplementary file 1 [file animals-14-01849-s001.zip › The list of supplementary.pdf]

Figure 1 : Gel electrophoresis showing the PCR amplification of markers 22276 (*N. quadranus*) and 291467 (*Q. yei*).
Figure 2: Sex determination in three frogs.
File S1 : Specimen information and location data
Figure S1 : Gel electrophoresis showing the PCR amplification of markers 446253 (*N. quadranus*).
Figure S2 : Mapping of the sex-linked markers of *N. quadranus* and *Q. yei* to the genome of *Q. spinosa*
Figure S3 : The mapping of sex-linked locus of *N. quadranus* and *Q. yei* on *AR* gene of *G. rugosa*.
Table S1 : Quality summary of *Q. yei* and *N. quadranus* by GBS sequencing.
Table S2 : Detailed information of all putatively sex-linked markers screened from *N.quadranus* and *Q.yei*.
Table S3 : Confirmed sex-linked makers after the elimination of false positives
Table S4 : Primer sequences of sex-linked markers isolated from *N. quadranus* and *Q. yei.*
Table S5 : The results of mapping sex-linked markers on to the genome of *Q. spinosa*.
Table S6: The results of mapping sex-linked markers on to the genome of *N. parkeri*.
Table S7: Details of sequencing datasets for *N. quadransua* and *Q. yei* analysesed.
Table S8: Blast results of candidate sex-determining genes to the reference genome.
Table S9: Mapping the sex-linked loci to the alignment region of *DMRT1* in the genome of *Q. spinosa*.
Table S10: Mapping the sex-linked loci to the alignment region of *DMRT1* in the genome of *N. parkeri*.
